# A Case of Midgut Volvulus Associated with a Jejunal Diverticulum

**DOI:** 10.1155/2017/3173875

**Published:** 2017-12-19

**Authors:** Joseph Gutowski, Rachel NeMoyer, Glenn S. Parker

**Affiliations:** ^1^Rutgers Robert Wood Johnson Medical School, Piscataway Township, NJ 08854, USA; ^2^Rutgers Robert Wood Johnson Medical School, New Brunswick, NJ 08901, USA; ^3^Jersey Shore University Medical Center, Neptune City, NJ 07753, USA

## Abstract

Midgut volvulus in adults is a rare entity that may present with intermittent colicky abdominal pain mixed with completely asymptomatic episodes. This small bowel twist may result in complications of obstruction, ischemia, hemorrhage, or perforation. With a midgut volvulus, complications may be life-threatening, and emergent surgical intervention is the mainstay of treatment. This current case involves an 80-year-old woman with intermittent abdominal pain with increasing severity and decreasing interval of time to presentation. A CAT scan revealed mesenteric swirling with possible internal hernia. A diagnostic laparoscopy followed by laparotomy revealed a midgut volvulus, extensive adhesions involving the root of the mesentery, and a large jejunal diverticulum. The adhesions were lysed enabling untwisting of the bowel, allowing placement of the small bowel in the correct anatomic position and resection of the jejunal diverticulum. This is a rare case of midgut volvulus with intermittent abdominal pain, associated with jejunal diverticulum managed successfully. A midgut volvulus should be considered in the differential diagnosis of a patient who present with a small bowel obstruction secondary to an internal hernia, especially when a swirl sign is present on the CAT scan.

## 1. Introduction

In the adult patient population, small bowel obstruction is a relatively common diagnosis. However, obstruction due to small bowel volvulus is quite rare, and most cases have been documented in newborns. Current literature suggests the annual incidence of small bowel volvulus to be 1.7–5.7 per 100,000 adults in Western countries [1]. Most commonly, it is attributed to congenital abnormalities or prior abdominal surgeries [1]. If left untreated, ischemia and subsequent infarction may ensue [2]. Here, we present a case of a patient undergoing an exploratory laparotomy for recurrent abdominal pain and imaging suggestive of an obstructive pathology who was found to have small bowel volvulus with a diverticulum.

## 2. Case Report

**Figure 1:**
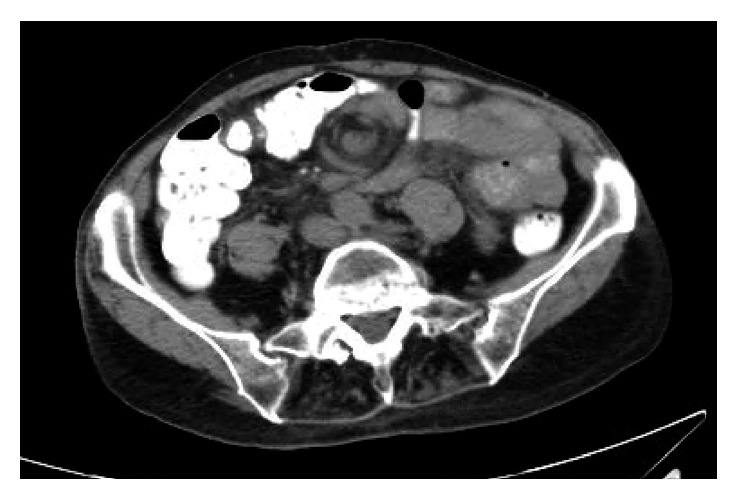
Axial view of CAT scan. Mesenteric swirl sign.

The patient is an 80-year-old female with a history of recurrent abdominal pain. Permission was obtained from the patient to allow discussion and publication of his case. The patient had a history of coronary artery disease, atrial fibrillation (on anticoagulation), multiple cerebral vascular accidents, chronic obstructive pulmonary disease, an aortic and mitral valve replacement, and a prior hysterectomy. The patient was noted to have presented to the hospital three times in the prior month with similar complaints of vague, diffuse abdominal pain that would last a few hours and resolve. The patient did note nausea and vomiting with these episodes. The patient re-presented to the emergency room, where a CAT scan was performed (Figure 1) which showed mesenteric swirling secondary to possible internal hernia. The patient underwent a small bowel follow through with gastrografin, which demonstrated no abnormalities. The patient then underwent an obstructive series that was also noted to be normal. The patient's symptoms, however, did not subside. Due to the chronicity and the unresolving symptoms, the patient was brought to the operating room after a long discussion of possible outcomes with the patient.

**Figure 2:**
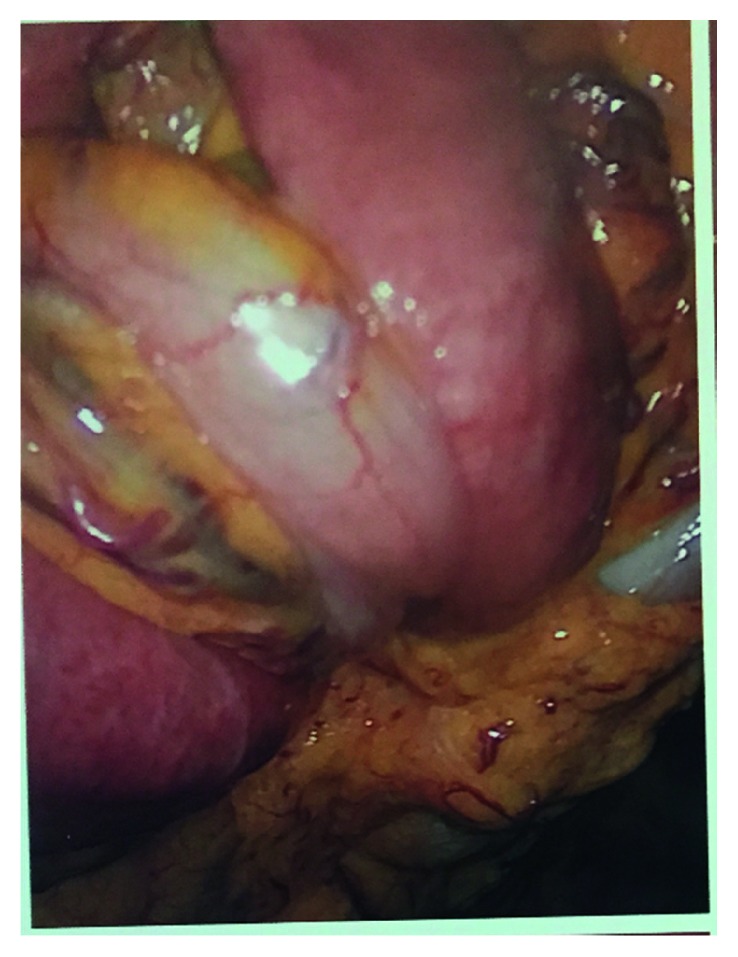
Laparoscopic view of adherent small bowel.

**Figure 3:**
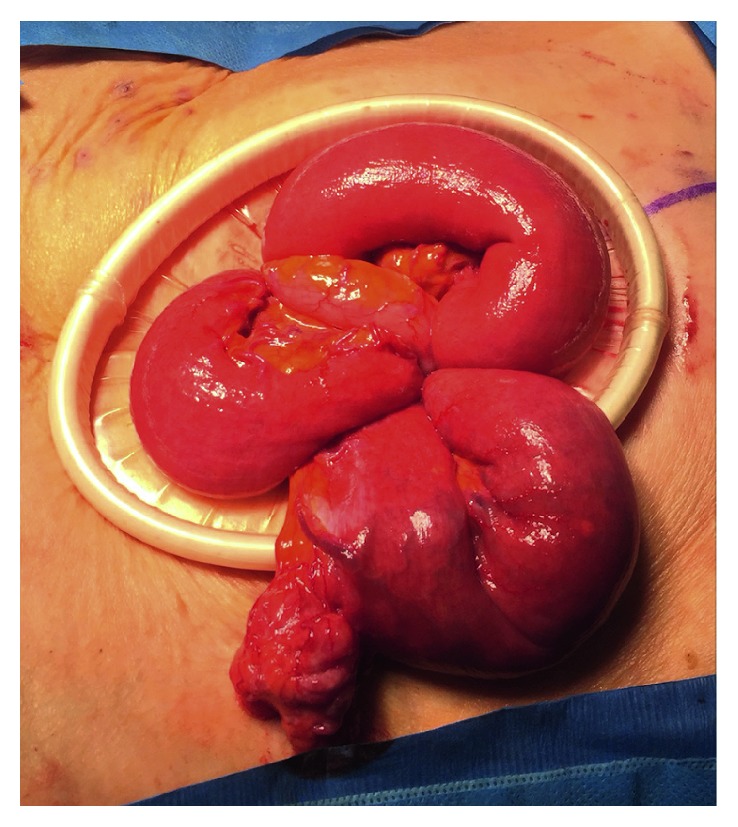
Open view of volvulized small bowel.

The operation was started laparoscopically but was soon converted to an open laparotomy due to a large mass of small bowel swirled onto itself that was adhered together (Figure 2). In mobilizing the small bowel, blunt dissection was used to lyse adhesions and untwist the mesentery. The small bowel mesentery was observed, and no mesenteric defects were noted. It was seen that the mesentery had swirled and twisted upon itself (Figure 3). While running the bowel, a large, proximal jejunal diverticulum was present. The diverticulum was excised using a 60 mm stapler. The bowel was run from the ligament of Treitz down to the ileocecal valve with no mesenteric defects noted, and all bowel was viable. The small bowel was returned to the abdomen. The patient did well postoperatively and was seen in follow-up without complication.

## 3. Discussion

Small bowel obstruction is a fairly common diagnosis in the adult population of the United States, with the most common causes being postoperative adhesions, masses, and hernias. Patients typically experience severe and intermittent abdominal pain, nausea, vomiting, and the inability to pass stool or flatus. A small bowel volvulus can occur if a portion of the bowel twists on itself and its mesentery, effectively causing an obstruction. If the obstruction is a closed-loop obstruction, which includes small bowel volvulus, the CAT scan can demonstrate a swirl sign. This finding represents mesenteric soft-tissue and fat attenuation with adjacent loops of bowel surrounding the rotated intestinal vessels [3]. A relatively recent study suggested that the swirl sign has a sensitivity of 64% and a PPV of 21% in diagnosing a small bowel volvulus [3].

The differential diagnosis for a patient presenting with symptoms of a small bowel obstruction may include adhesions, hernia, neoplasm, intussusception, small bowel hematoma, or pathology relating to the patency of the lumen such as a bezoar or a gallstone [4]. Volvulus accounts for only 4 to 15% of mechanical small bowel obstructions in the United States and Western Europe [5]. More common is volvulus of the large intestine, specifically at the sigmoid, which makes up 70 to 80% of large intestine cases, followed by the cecum, which makes up 10 to 20% of large intestine cases [2].

Small bowel volvulus may be classified as primary or secondary. Primary volvulus occurs in patients with a virgin abdomen who possess no anatomic abnormalities that would predispose them to develop a volvulus [2]. These represent 10 to 22% of all volvulus cases in the Western world [2]. Secondary volvulus is much more common in the United States. It occurs in patients who have congenital or acquired pathology of the abdomen such as adhesions, tension bands, or anatomic malformations [2]. Diverticulum of the small bowel, such as the one seen in our patient, has also been associated with the development of volvulus, as one study found the incidence of small bowel diverticulum in patients with small bowel volvulus to be as high as 35% [6].

The primary determinant in reducing the morbidity and mortality of small bowel volvulus is early diagnosis and treatment [2]. If the obstruction becomes advanced, vascular compromise can occur thus increasing the risk of intestinal necrosis and subsequent intestinal perforation. Unfortunately, it is difficult to accurately diagnose ischemic bowel on CAT scan [4]. The best indicators of gangrenous bowel are peritoneal signs, a palpable mass, fever, and leukocytosis [7]. In addition to the metabolic alkalosis that can result from excessive vomiting, a metabolic acidosis can occur if the bowel is ischemic due to the accumulation of lactic acid [8]. Early surgical intervention remains the best form of treatment for small bowel volvulus. Manually untwisting the volvulus may be sufficient if the bowel appears healthy and viable after the maneuver. However, gangrenous bowel must be resected to minimize the risk of infection and perforation [2].

This is a rare case of midgut volvulus with intermittent abdominal pain, associated with jejunal diverticulum managed successfully. A midgut volvulus should be considered in the differential diagnosis of a patient who presents with a small bowel obstruction secondary to an internal hernia, especially when a swirl sign is present on the CAT scan to minimize morbidity and possible mortality of the patient.
